# C-854-07. Improving Nutrition for Patients with Severe Burns Utilizing a Nurse-Driven Volume-Based Enteral Feeding Protocol

**DOI:** 10.1093/jbcr/irag033.083

**Published:** 2026-04-06

**Authors:** Pamela E Garlinghouse, Molly McNett, Todd E Tussing, Kelly Casler

**Affiliations:** The Ohio State Comprehensive Burn Center, Columbus, OH; Sacred Heart University, Columbia, MO; The Ohio State University, Columbus, OH

## Abstract

**Introduction:**

The importance of nutritional support in severely burned patients (greater than 20% total body surface area) is well documented, but the provision of adequate nutrition is frequently suboptimal. Enteral nutrition (EN) helps to meet these nutritional goals. A nurse-driven volume-based feeding (VBF) approach focuses on the goal volume a burn patient receives in a 24-hour period. The purpose of the initiative was to improve successful utilization of VBF for hospitalized burn patients who require EN.

**Methods:**

This quality improvement initiative at our 38-bed comprehensive Surgical, Trauma, and Burn Intensive Care Unit is located within a 900-bed academic Level 1 Trauma and American Burn Association verified adult Burn Center tracked the performance of a nurse-driven protocol for all severely burned patients who required EN. Implementation was guided by the Plan-Do-Study-Act (PDSA) framework, and three key strategies were used to support the primary goal of increasing nutritional delivery of prescribed nutrition to evidence-based (EB) recommendations of greater than 80%. First, a reactivated order set was updated and customized to include a cascading VBF ordering tab that allows for the tailoring of nutrition specific to the unit and patient for an easier, more customized delivery. Second, nursing education included expert led micro-education combined a “Snack and Learn” presentation, quiz, program feedback and CE credit. Third, adoption and fidelity of the VBF protocol were evaluated using a customized auditing checklist with real time follow up.

**Results:**

Over a 12-week observation period protocol adoption and fidelity were tracked for the admitted six severely burned patients. The number of days patients received VBF ranged from 2 to 15, with an average of 8.6 days. One patient expired during the observation period, which resulted in a total of 5 patients with full audits. Upon audit completion, findings indicated a high utilization of the VBF protocol This included 100% for ordering, 100% for documentation of required components, and 100% of patients meeting nutritional goals of 80%. The implementation of an electronic VBF protocol has increased the number of patients who received target nutritional goals. This successful implementation continues and has been adopted as a permanent protocol.

**Conclusions:**

The use of a standardized VBF protocol, combined with education, auditing, and ongoing evaluation can be an effective method to align current practices with EB recommendations. Providing education and real time follow-up can improve the process.

**Applicability of Research to Practice:**

The use of a nurse-driven standardized protocol, incorporating education, auditing, and ongoing evaluation can be an effective method to align current practices with evidence-based recommendations for burn patients.

**Funding for the Study:**

N/A.

